# Prevalence of metabolic syndrome and its correlated factors among children and adolescents of Ahvaz aged 10 – 19

**DOI:** 10.1186/2251-6581-13-53

**Published:** 2014-04-28

**Authors:** Homeira Rashidi, Seyed Peyman Payami, Seyed Mahmoud Latifi, Majid Karandish, Armaghan Moravej Aleali, Majid Aminzadeh, Kuroush Riahi, Marzieh Ghasemi

**Affiliations:** 1 Health research Institute, Diabetes Research Center, Ahvaz Jundishapur University of Medical Sciences, Ahvaz 61357-15794, Iran; 2 Nutrition and Metabolic research center, Ahvaz Jundishapur University of Medical Sciences, Ahvaz, Iran

**Keywords:** Metabolic syndrome, Prevalence, Children, Adolescents, Cardiovascular risk factor, Iran

## Abstract

**Background:**

Population-based studies for prevalence of metabolic syndrome (M.S) in children and adolescents are relatively rare. The aim of this study was to assess the Prevalence of M.S and correlated factors among children and adolescents aged 10 to 19 years in Ahvaz.

**Methods:**

In this descriptive-analytical population- based study, 2246 children and adolescents, 10–19 years old (1113 male and 1133 female) in Ahvaz, were evaluated. Anthropometry, biochemical measurement and blood pressure (BP) were assessed. Modified ATP III criteria 2005 were used for M.S. definition. Center for disease and Control preventions (CDC) percentile were applied to define cut off points of waist circumference and BP.

**Results:**

Prevalence of M.S. was 9% (95% CI: 8-10%) with prevalence in male 11% (95% CI: 10-12%) and female 7% (95% CI 6-8%). Among individuals with M.S, triglyceride (TG) and decreased high density lipoprotein (HDL) cholesterol levels were the most common components (33.5% and 24.1%, respectively). Prevalence of M.S was higher in overweight persons comparing to participants with at risk and normal weight group (in male: 24.1%, 14.3% and 9.9% respectively P = 0.0001), (in female: 22.6%, 18.3% and 4.5% respectively P = 0.0001). Among the correlated factors of M.S age (P = 0.0006), sex and BMI (P = 0.0001) had significant differences between subjects with and without M.S. whereas there was no significant difference between two groups in ethnicity, history of breast fed, birth weight neonatal ICU admission, maternal history(GDM, gestational HTN, Parity) and family history of HTN, obesity and DM (P > 0.05).

**Conclusion:**

This study shows high prevalence of M.S in Children and Adolescents in south west of Iran (Ahvaz) especially in overweight persons.

## Introduction

Since early 1960s, an association between obesity and elevated level of triglycerides (TG), decreased level of high-density lipoprotein (HDL) cholesterol, hyperinsulinemia, impaired glucose tolerance, high blood pressure (BP) and cardiovascular disease, (CVD) has been discussed. For first time it was introduced in 1988 by Reaven et al. as metabolic syndrome (M.S) [1]. M.S is the risk factor of all cause and C.V. mortality in adults with and without type 2 diabetes [2]. According to some studies, M.S could be originated from embryonic period [3,4]. Although the prevalence of M.S and its risk factors have been widely studied in adults, limited studies are available in children and adolescents so that there is no clear definition of M.S in this age group. Therefore more studies are required to provide a better definition of M.S in children and adolescents. Currently, the same risk factors of adults are used in this age group based on age and gender percentiles from global or rational data or study specific distributions. Based on limited studies 4.2% of the American children and adolescents who participated in the NHANES III 1988-1994 had M.S. [5]. That increased to 6.2% in NHANES 1999–2000 [6]. As the prevalence of overweight in American children and adolescents increase [7], the prevalence of M.S is likely to rise, such that above study showed that 29% and 32% of overweight subjects had M.S., respectively. As well, a recent study in Tehran revealed that the prevalence of overweight in Iranian children and adolescents was high with an estimated prevalence of 21% [8]. On the other hand, in another study in Tehran, the prevalence of M.S in children and adolescents was estimated about 10% based on modified ATPIII criteria [9]. These studies indicated that the prevalence of M.S is significantly higher in more obese subjects. Recently, the prevalence of M.S has been reported among Mexican [10], Tunisian [11], Indian [12] and Chinese [13] children and adolescents. Different genetic, racial [14] and environmental factors like lifestyle, change of lifestyle from rural to urban, physical activity and socioeconomic conditions play significant role in the prevalence of M.S. Therefore, this study was designed to determine the prevalence of M.S and its risk factors among children and adolescents of Ahvaz aged 10–19 years old.

## Materials and methods

This descriptive-analytic study performed in Ahvaz, capital city of Khuzestan province (South West of Iran) in 2009–2011. Of 25 health centers, 6 ones were selected randomly by multi-steps cluster sampling method. Informed written consent was obtained from subjects aged 10–19 and parents of subjects under the age of 19. A questionnaire including age, sex, ethnicity, birth weight, history of breast feeding, neonatal ICU admission, history of pregnancy in participant’s mother [gestational diabetes mellitus (GDM), gestational hypertension and parity number], family history of diabetes mellitus (DM), hypertension (HTN) and obesity was filled for each subject. In this study after excluding subjects taking medication that would affect serum lipids, blood pressure and carbohydrate metabolism and subjects with history of chronic disease, of heart, lung, kidney and liver and chronic diarrhea and hospitalization during three months ago, individual with full relevant data were included. Blood pressure was measured twice at least 30 minutes apart by an appropriate mercury sphygmomanometer after 15 minutes rest in a sitting position. The mean of the two readings was recorded as individual blood pressure.

Waist circumference was measured at the midpoint of the lowest rib and iliac crest over light clothing at the end of exhalation using a tape measure. Heights and weights were measured, using a tape measure in a standing position with bare feet and seca scales with minimum possible clothing, respectively. Body mass index (BMI) [weight (Kg)/Height (m) 2] was calculated for each subject. Blood samples were drawn after a 12- hour overnight fasting. About 30–45 minutes after sampling, the samples were centrifuged by 2500–3000 rpm for 10 minutes; sera were stored in refrigerator and then sent to laboratory. Fasting blood sugar (FBS), TG, total cholesterol and HDL cholesterol were measured by enzyme-calorimetric method using Pars Azmoon kits (with biotechnical instruments type BT-3000 Germany). Modified ATP III criteria 2005 [5,6] were used to define M.S as follows:

1- Abdominal obesity (waist circumference ≥ the age and sex specific 90^th^ percentile using C.D.C percentiles)

2- Elevated BP(systolic and/or diastolic blood pressure ≥the age and sex -specific 90^the^ percentile using C.D.C percentiles except for 18 and 19 years old subjects, for whom the cut off values of ≥130 and/or ≥85 mmHg for systolic and diastolic blood pressure were used, respectively.

3- HDL-cholestrol≤40

4- TG≥110 mg/dl

5- FBS≥100mg/dl

Subjects with 3 or more characteristics of the above components were categorized as M.S.

Overweight, at risk for Overweight and normal weight were defined based on the study specific percentile curves of BMI for age and sex as ≥95^th^ percentile, ≥85 to <95^th^ percentile and <85^th^ percentile, respectively.

Descriptive statistics were used to prepare graphs and tables. Chi -Square test was applied to compare ratios and multivariate logistic regression analysis was used to determine the potential determinants of M .S.

P < 0.05 was considered as significant. All data were analyzed by SPSS software version 19.

## Results

**Figure 1:**
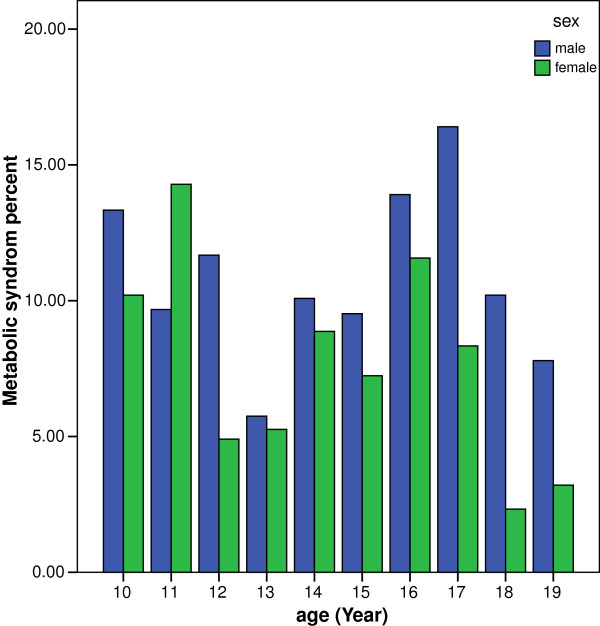
Prevalence of Metabolic syndrome in different sex and age groups.

A total of 2246 children and adolescents (1120 boys, 1139 girls) aged 10–19 participated in our investigation. Baseline characteristics of study population according to sex is shown in Table 1. According to the modified ATPIII criteria,the prevalence of M.S was 9% (95% CI: 0.08-0.10). The prevalence of MS in boys [11% (95% CI: 10-12%)] was significantly higher than girls [7% (95% CI: 6-8%)], (P==0.001) (Table 2). In both gender, the prevalence of MS was different between age groups. The prevalence of MS decreases approximately after the age of 10 to 11 while it increases after the age 14 and highest rate was observed at ages 16 to17. In boys, the relationship between the prevalence of M.S and increase of age was insignificant (P = 0.403) but it was significant in girls (P = 0.010) (Figure 1).

**Table 1:** Basic characteristics of the study population according to sex

**Variable**	**Male**	**Female**	**P value**
**Age (year)**	14.63 ± 2.58	15.27 ± 2.74	0.0001
**Height (cm)**	162.83 ±v13.82	151.81 ± 9.32	0.0001
**Weight (Kg)**	52.39 ± 14.69	50.99 ± 12.39	0.015
**BMI (Kg/m**^**2)**^	19.75 ± 4.43	20.83 ± 4.20	0.0001
**Waist circumference (cm)**	70.69 ± 11.23	68.19 ± 9.82	0.0001
**HDL (mg/dl)**	53.77 ± 12.18	55.62 ± 11.77	0.0001
**TG (mg/dl)**	111.62 ± 67.07	100.52 ± 57.66	0.0001
**SBP (mm Hg)**	106.58 ± 11.25	106.32 ± 10.99	0.574
**DPB (mm Hg)**	60.65 ± 10.20	64.85 ± 9.68	0.0001
**FBS (mg/dl)**	91.85 ± 12.45	89.16 ± 12.45	0.012

**Table 2:** Prevalence of M.S in different sex and age groups

**Age (year)**	**N**	**No. of M.S (%) Total**	**N**	**No. of M.S (%) (Male)**	**N**	**No. of M.S (%) (Female)**
10	94	11 (11.7)	45	6 (13.3)	49	5 (10.2)
11	208	24 (11.5)	124	12 (9.7)	84	12 (14.3)
12	239	21 (8.8)	137	16 (11.7)	102	5 (4.9)
13	163	9 (5.5)	87	5 (5.7)	76	4 (5.3)
14	243	23 (9.5)	119	12 (10.1)	124	11 (8.9)
15	299	25 (8.4)	147	14 (9.5)	152	11 (7.2)
16	272	35 (12.9)	151	21 (13.9)	121	14 (11.6)
17	236	30 (12.7)	128	21 (16.4)	108	9 (8.3)
18	227	13 (5.7)	98	10 (10.2)	129	3 (2.3)
19	265	12 (4.5)	77	6 (7.8)	188	6 (3.2)
Total	2246	203 (9)	1113	123 (11.1)	1133	80 (7.1)
P Value		0.0006		0.0403		

When examined by BMI category, overweight group had a higher prevalence of MS than at risk for overweight and normal weight groups (P = 0.0001) (Table 3).

**Table 3:** Prevalence of metabolic syndrome in different body weight groups

	**BMI status(percentile)**	**No. of subjects (%)**	**Subjects with M.S (%)**	**P value**
**Total**	Normal (<85)	1911 (85.1)	7.2	0.0001
	At risk (85-95)	227 (10.1)	16.3	
	Overweight (≥95)	108 (4.8)	26.9	
**Male**	Normal (<85)	946 (85.1)	9.9	0.003
	At risk (85-95)	112 (10.1)	14.3	
	Overweight (≥95)	54 (4.9)	24.1	
**Female**	Normal (<85)	946 (85.1)	4.5	0.0001
	At risk (85-95)	115 (10.2)	18.3	
	Overweight (≥95)	54 (4.8)	29.6	

In this study, 46% and 5% of the participants with a waist circumference ≥90^th^ percentile and <90% percentile had M.S respectively (P = 0.0001). The mean of waist circumference between two genders showed significant difference (P = 0.0001). The prevalence of each component of M.S in subjects with M.S was: abdominal obesity 10.3% elevated BP 22.1%, low HDL-cholesterol 24.1%, high TG 33.5% and high FBS 16.4% (Table 4). In subjects with M.S., 81.8% had three components of M.S, 16.7% had four and 1.5% had five ones. In the whole study population, 36.5% were normal (no components of M.S), 34.6% had one. 19.9% Two, 7.3% three, 1.5% four and 0.1% had five components.

**Table 4:** Prevalence of individual component of metabolic syndrome according to age and sex among subjects with metabolic syndrome

**Age (year)**	**Abdominal obesity (%)**	**TG ≥ 110 (%)**	**HDL ≤ 40 (%)**	**BP ≥ 130/85 (%)**	**FBS ≥ 100 (%)**
**Total population**					
**10**	9.7	34	18.1	36.2	21.3
**11**	11.4	34.6	17.3	34.6	19.2
**12**	10.3	35.2	20.9	24.3	18.6
**13**	9.4	37.7	19	16	14.2
**14**	10.2	33.3	27.2	21.4	25.1
**15**	9.6	36.7	27.8	26.1	11.2
**16**	10.7	31.8	30.5	36.4	15.4
**17**	10	40.6	28	25.4	15
**18**	11.1	26.3	21.1	3.1	12.5
**19**	10	26.1	23.1	3.8	14.9
**Total**	10.3	33.5	24.1	22.1	16.4
**Males**					
**10**	11.1	31.1	11.1	24.4	22.2
**11**	9.7	33.9	15.3	33.9	18.5
**12**	11.1	34.8	19.7	35	21.3
**13**	9.3	37.2	14.9	16.1	17.4
**14**	9.5	34.5	26.1	24.4	30.3
**15**	9.7	40.3	31.3	27.9	13.9
**16**	10.6	36.1	37.1	35.8	15.6
**17**	9.6	48.4	32.8	24.2	15.6
**18**	11.5	32	35.7	5.1	17.5
**19**	10.4	36.8	36.4	3.9	23.7
**Total**	10.2	37.1	27.1	25	19.1
**Females**					
**10**	8.3	36.7	24.5	46.9	20.4
**11**	14.1	35.7	20.2	35.7	20.2
**12**	9.2	35.6	22.5	9.8	14.9
**13**	9.5	38.2	23.7	15.8	10.5
**14**	10.8	23.3	27.2	18.5	20.2
**15**	9.5	33.3	24.3	38.2	8.7
**16**	10.7	26.7	22.3	37.2	15
**17**	10.5	31.1	22.2	26.9	14.2
**18**	10.9	22	10.1	1.6	8.7
**19**	9.8	21.6	17.6	3.7	11.4
**Total**	10.3	29.9	21.1	19.3	13.6

TG: Trigliceride, HDL-D: high density lipoprotein cholesterol, BP: blood pressure, FBS: fasting blood suger.

Factors that can affect the prevalence of M.S are seen in Table 5. Among the correlated factors of M.S age (P = 0.006), sex and BMI (P = 0.0001) had significant differences between subjects with and without M.S. whereas ethnicity, breast feeding, birth weight, neonatal ICU admission, maternal history (GDM, Gestational HTN, Parity no) and family history of HTN, obesity and D.M had no differences (P > 0.05).

**Table 5:** Risk factors of metabolic syndrome among children and adolescences

	**B**	**S.E**	**P value**	**OR**	**Confidence interval (CI)**
**Sex (M/F)**	0.643	0.178	0.000	1.903	1.341-2.700
**Ethnicity (Arab/Fars)**	-0.019	0.174	0.914	0.981	0.698-1.380
**Birth weight**	0.207	0.139	0.135	1.230	0.937-1.614
**Neonatal nutrition (breastfeeding/other)**	0.003	0.617	0.991	1.003	0.630-1.596
**ICU Admission**	0.356	0.508	0.483	1.428	0.528-3.863
		**Family history**			
**Obesity**	0.087	0.183	0.647	1.087	0.760-1.555
**Diabetes**	0.430	0.223	0.054	1.523	0.993-2.380
**HTN**	0.278	0.226	0.204	1.333	0.856-2.076
		**Maternal history**			
**Parity no.**	0.045	0.042	0.286	1.046	0.963-1.136
**GDM**	-0.305	0.508	0.548	0.737	0.273-1.994
**Gestational HTN**	0.362	0.360	0.314	1.437	0.709-2.910
		**BMI**			
**BMI 85-95/BMI<85**	0.970	0.243	0.001	2.639	1.640-4.248
**BMI≥95/BMI<85**	1.645	0.303	0.001	5.180	2.859-9.387
		**Age (Year)**			
**11/10**	-0.381	0.429	0.374	0.683	0.295-1.583
**12/10**	-0.326	0.415	0.432	0.722	0.320-1.628
**13/10**	-0.945	0.490	0.054	0.389	0.149-1.017
**14/10**	-0.556	0.426	0.192	0.573	0.249-1.322
**15/10**	-0.672	0.420	0.109	0.511	0.224-1.163
**16/10**	-0.244	0.403	0.546	0.784	0.356-1.728
**17/10**	-0.493	0.422	0.243	0.611	0.267-1.397
**18/10**	-1.196	0.473	0.011	0.302	0.120-0.763
**19/10**	-1.875	0.519	0.000	0.153	0.056-0.424

S.E: Stanndard error.

B: coefficient of variable.

O.R: Odd ratio.

## Discussion

In this study, the prevalence of M.S among children and adolescents aged 10–19 was 9% based on the modified ATPIII 2005 criteria which was significantly higher in boys than girls (P = 0.001). The prevalence of M.S in our study was similar to the study of Esmailzadeh et al. [9] in Tehran based on same criteria. In that report, prevalence of M.S was not significantly different between boys and girls and study- specific distributions to define thresholds of risk factors were used. In another study in Tehran by Chiti et al. [15], the prevalence of M.S among children and adolescents aged 10–19 was 9.5% based on the same criteria which was higher in boys than girls. The result of that study was consistent with ours. Kelishadi et al. [16] conducted another study in students aged 6–18 in Isfahan that showed the prevalence of M.S as 14%, indicating a much difference with above- mentioned studies.

Other studies in different cities of Iran [17-19], not population- based, could not reflect the whole society. Population - based epidemiologic studies in other countries have shown various prevalence rates. In the U.S, the prevalence of M.S among children and adolescents has been reported 3.1% to 12.7% with different definitions and cut off points [20]. These studies have revealed that the prevalence of M.S. is increasing by time in this age group. The investigation of Herrabi et al. [11] in Tunisia showed that the prevalence of M.S. is 4% in urban regions based on the modified -ATPIII criteria. Overweight and obesity were the most common components of the syndrome. In another study in north India, Singh et al. [13] reported the prevalence of M.S to be 4.2% using modified ATPIII criteria. That study again confirmed that the prevalence of M.S is higher in at risk for overweight and overweight persons. A study in Mexico by Rodrignes -Moran et al. [11] using modified ATP III criteria showed prevalence of M.S. to be 6.5% while it was 4.5% by WHO criteria. As well, an investigation in China by Liet of [13] using the criteria proposed by Ferranti et al. [20], reported the prevalence of M.S to be 3.7%. According to that study, it is estimated that 3 million children and adolescents have M.S in China. The lack of a comprehensive and clear definition of M.S in children and adolescents could explain to some extent. The difference of prevalence rates reported in various studies throughout the world. On the other hand, the factors like various prevalence of overweight, race, genetic factors, change in lifestyle from rural to urban, physical activity level and socioeconomic condition could be considered. Like other studies in Iran [8,19,21], our investigation showed a higher prevalence of M.S. among at risk for overweight and overweight children and adolescents in both genders.

On the other hand, the prevalence of overweight in Iranian children has been reported to be up to 8%. The change of nutritional style in Iranian children and adolescents is probably an important reason for the uprising problem of overweight and M.S. As well, some evidences are available that indicates decrease in physical activity of Iranian children and adolescents [22,23]. Moreover, recent epidemiological studies show a parallel increase in type 2 diabetes mellitus in children/adolescents and obesity [24,25]. Unfortunately, the rise in prevalence of M.S. and type 2 D.M in children and adolescence lead to an increase in correlated complications in young adults, including early C.V.D [20].

The current study has several strengths. It was a population- based study with a relatively large sample size. Moreover numerous correlated factors were assessed. One limitation was the definition of M.S. limitations of such a definition for children and adolescents has been discussed previously [5,20,26]. Cross sectional nature of this study was the other limitation that did not allow us to make causal inference. Our study provides evidences showing a high prevalence of M.S. among Iranian children and adolescents, especially in overweight subjects. In the future, large prospective studies should be conducted to confirm the association between above- mentioned factors and M.S.

## Conclusion

Among the factors correlated to M.S., age, sex and BMI were significantly different between subjects with and without M.S. whereas ethnicity, breast feeding, birth weight, neonatal ICU admission, maternal history (GDM, gestational hypertension, parity number) and family history of hypertension, obesity and diabetes mellitus did not show significant differences.

## Competing interests

The authors declare that they have no competing interests.

## Authors’ contributions

HR, MA and MK designed the study. SPP drafted the manuscript. SML has done the statistical analysis. AMA, KR and MGh have done data collection, references search, writing and editing manuscript. All authors read and approved the final manuscript. Thanks to all authors for their support and help in this study.
